# Short-term histopathological effects of direct peripheral nerve exposure to AH Plus® Bioceramic Sealer in rats: an exploratory assessment of Stemregen® supplementation

**DOI:** 10.2340/biid.v13.46483

**Published:** 2026-07-08

**Authors:** Haydar İrfan Yücel, Mevlüt Sinan Ocak, Ebru Gökdere, Serkan Dundar, İbrahim Hanifi Özercan

**Affiliations:** a Department of Endodontics, Faculty of Dentistry, Firat University, Elazıg, Turkey; b Department of Physiology, Faculty of Medicine, Firat University, Elazıg, Turkey; c Department of Periodontology, Faculty of Dentistry, Firat University, Elazıg, Turkey; d Department of Pathology, Faculty of Medicine, Firat University, Elazıg, Turkey

**Keywords:** dietary supplements, nerve damage, premixed bioceramic, sciatic nerve

## Abstract

**Background:**

Extrusion of root canal sealers into neurovascular tissues can result in rare but serious neurological complications, including paresthesia and neuropathic pain. This study aimed to evaluate the histopathological effects of direct peripheral nerve exposure to AH Plus® Bioceramic Sealer and to assess the influence of Stemregen® supplementation on histopathological tissue responses.

**Materials and methods:**

Forty-eight male Sprague-Dawley rats were randomly assigned to four groups (* n* = 12): Control, Supplement only, Bioceramic only, and Bioceramic + Supplement. Sterile polyethylene tubes containing AH Plus® Bioceramic Sealer were applied in contact with the sciatic nerve in the relevant groups. Stemregen® was administered orally at 300 mg/kg/day for 15 days. At the end of the experiment, nerve tissues were harvested and examined histologically. Nerve tissues were evaluated histologically for axonal and myelin degeneration, inflammatory infiltration, fibrosis, and perineurial changes using semiquantitative scoring, and intergroup comparisons were performed using nonparametric statistical tests (Kruskal–Wallis test followed by Dunn’s post hoc analysis).

**Results:**

Severe axonal degeneration, inflammation, and fibrosis were observed in the Bioceramic group compared to controls (* p* < 0.05). The Bioceramic + Supplement group showed lower histological damage scores than the Bioceramic-only group; however, these differences were not statistically significant (* p* > 0.05).

**Conclusions:**

These findings indicate that direct exposure to a bioceramic root canal sealer is associated with histopathological alterations in peripheral nerve tissue, whereas systemic administration of Stemregen® was associated with lower injury scores without achieving statistical significance.

## Graphical abstract

**Figure:**
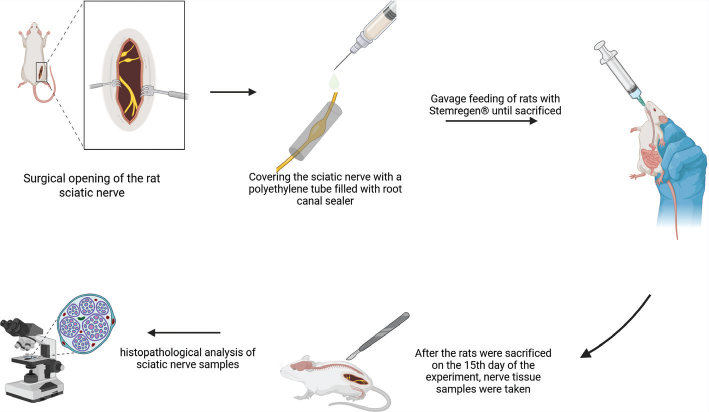
GRAPHICAL ABSTRACT

**KEY MESSAGES:**

1. Direct exposure of peripheral nerve tissue to AH Plus® Bioceramic Sealer was associated with significant histopathological degeneration and inflammatory changes in a rat sciatic nerve model; however, functional neural outcomes were not assessed.
2. Stemregen® supplementation was associated with lower histopathological injury scores compared with the bioceramic-only group although the differences were not statistically significant.
3. The findings emphasize the importance of preventing sealer extrusion near neurovascular structures and support the need for further translational studies on the neurobiological effects of calcium silicate–based sealers.

## Introduction

Although endodontic sealers are widely used in routine clinical practice, unintended apical extrusion into adjacent neurovascular structures may result in rare but potentially severe neurological complications, including paresthesia, hypoesthesia, and persistent neuropathic pain [1–5]. Such adverse outcomes are most commonly reported in the posterior mandible, where the proximity of root apices to the mandibular canal and the porous trabecular bone architecture facilitate the diffusion of extruded materials toward neural tissues [6]. The clinical severity of nerve injury is influenced by factors such as the volume of extruded material, duration of nerve contact, and the intrinsic biological properties of the sealer [7–9]. Depending on the extent of injury, management strategies range from conservative pharmacological approaches to surgical intervention in progressive or irreversible cases [10–13].

Bioceramic-based root canal sealers have gained widespread acceptance due to their favorable physicochemical and biological properties, including bioactivity, alkaline antimicrobial effects, and reported biocompatibility. These materials are primarily composed of calcium silicate phases, such as tricalcium silicate, which promote mineralization and tissue compatibility under standard conditions [14, 15]. AH Plus® Bioceramic Sealer (Dentsply Sirona, Ballaigues, Switzerland) is a premixed injectable formulation developed within this material class and has demonstrated satisfactory physicochemical performance and cytocompatibility in in vitro studies [16–18]. However, in vitro biocompatibility does not necessarily reflect biological behavior under conditions of direct neural exposure. Data regarding the in vivo neurotoxic potential of bioceramic sealers following accidental extrusion and direct contact with peripheral nerve tissue remain limited [19–24]. AH Plus® Bioceramic Sealer was selected as a representative injectable calcium silicate–based sealer with documented physicochemical stability and reported biological compatibility, allowing evaluation of neural tissue responses relevant to contemporary endodontic practice.

A recent in vitro neurocompatibility study suggests that endodontic materials may differ in their neuronal tolerance, highlighting the need for in vivo models to better understand neural tissue responses under direct contact conditions [25]. Recent experimental approaches to peripheral nerve injury have increasingly focused on modulating the inflammatory microenvironment rather than directly targeting neural regeneration. In this context, nutritional supplements that support endogenous repair mechanisms and exert systemic anti-inflammatory effects have been explored as adjunctive strategies [26–28]. Stemregen® (Biomics™, Incline Village, NV, USA) is a commercially available multi-component dietary supplement whose ingredients have individually been reported to possess anti-inflammatory or antioxidant properties in experimental settings. Its composition is presented in Table 1. Although several of its botanical constituents have demonstrated antioxidant and anti-inflammatory properties in experimental models, its potential influence on peripheral nerve tissue following chemical or material-induced injury has not been evaluated in vivo [29–37]. Therefore, Stemregen® was included in the present study as an exploratory adjunctive intervention to investigate whether supplementation might influence histopathological tissue responses following direct exposure of peripheral nerve tissue to a bioceramic sealer.

**Table 1:** Active ingredients and corresponding amounts contained in each capsule of Stemregen®.

Active ingredient	Amount
Sea buckthorn extract (30% proanthocyanidins)	250 mg
Blue-green algae (Aphanizomenon flos-aquae) extract	150 mg
Bladderwrack (Fucus vesiculosus) extract (20% phlorotannins)	125 mg
Notoginseng (Panax notoginseng) extract (10% notoginsenosides)	62.5 mg
Aloe vera gel powder (Stemaloe®)	50 mg
1^→^3 Beta-glucan (85%)	50 mg
Colostrum powder (transfer factor)	25 mg
Black pepper (Piper nigrum) extract	2.5 mg

The table summarizes the botanical and nutritional components of the supplement administered to the experimental groups during the study period.

Therefore, the present study aimed to evaluate the histopathological effects of AH Plus® Bioceramic Sealer following direct contact with peripheral nerve tissue using a rat sciatic nerve model and to explore whether systemic administration of Stemregen® could modulate inflammation-associated histopathological changes. It was hypothesized that direct exposure to the bioceramic sealer would induce neural tissue damage, while Stemregen® supplementation might influence these histopathological responses. The null hypothesis was that direct exposure to the bioceramic sealer would not result in histopathological nerve damage and that Stemregen® administration would have no modulatory effect on neural tissue responses.

## Material and methods

This study was prepared in accordance with the PRILE 2021 guidelines for the reporting of animal experiments in the field of Endodontics; the flow diagram summarizing the experimental process is presented in Figure 1. The study was conducted with the permission numbered 2023/16-04 dated 20.09.2023 from the Firat University Local Ethics Committee for Animal Experiments and was carried out in accordance with the ARRIVE guideline principles regarding the protection of laboratory animals. All rats used in the experiment were provided by the Firat University Experimental Research Center, and the experimental stages were carried out at the same center and at the Firat University Faculty of Medicine Pathology Laboratory.

**Figure 1:**
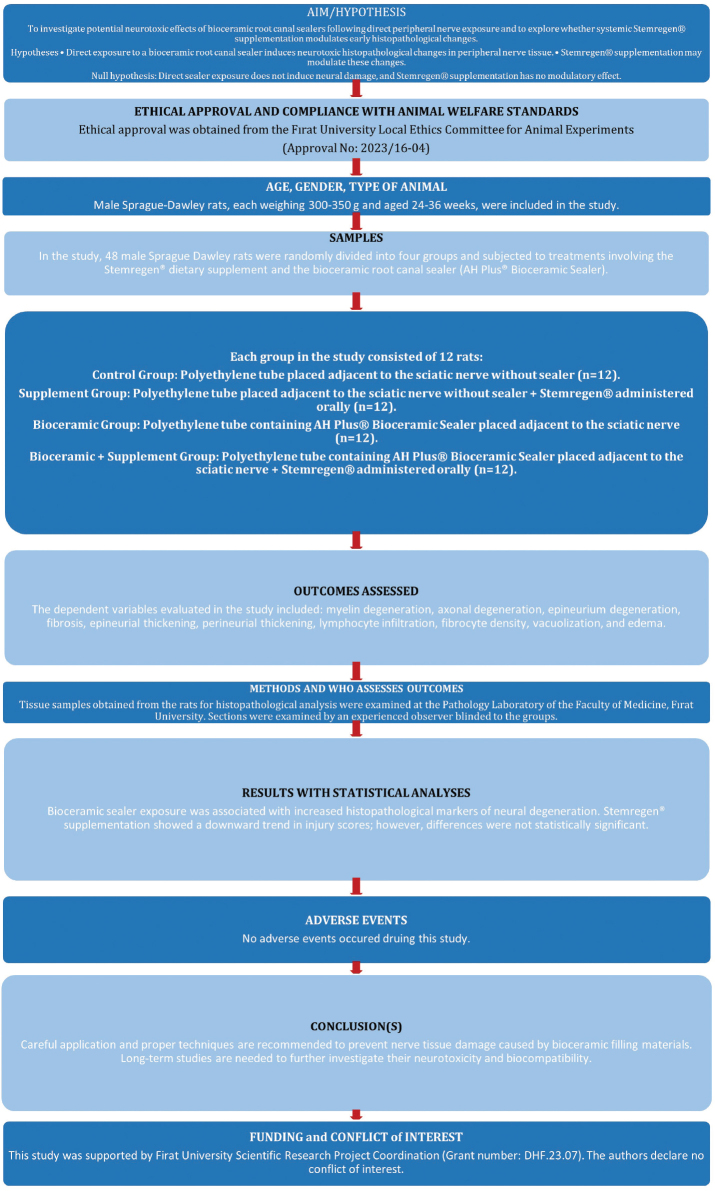
Flowchart summarizing the experimental design of the study (PRILE 2021).

### Determination of sample size and formation of experimental groups

The sample size was determined using GPower software (version 3.1.9.2; Heinrich Heine University, Düsseldorf, Germany). In line with previous experimental studies using rat sciatic nerve models to evaluate the neurotoxic effects of root canal sealers through histopathological assessment, where significant differences were detected with comparable or smaller group sizes, a medium effect size (* f* = 0.40) was assumed [20]. With an α error probability of 0.05 and a desired statistical power of 80% (1−β = 0.80), the minimum required sample size was calculated as 12 animals per group. Male Sprague-Dawley rats aged between 24 and 36 weeks and weighing approximately 300–350 g, provided by FUERC, were used. Rats were preferred in the study due to their genetic similarity to humans and their common use in experimental studies. Additionally, male rats were used to minimize potential confounding effects of hormonal fluctuations associated with female reproductive cycles. All subjects were kept during the experiment in polyethylene cages with unrestricted access to food and water, in rooms with special ventilation systems at 70% humidity and 22–24°C temperature. Animals were randomly assigned to the experimental groups using a random number generator as summarized in Table 2.

**Table 2:** Experimental groups and corresponding interventions in the rat sciatic nerve model.

Group	Number of animals (* n*)	Intervention
Control (C)	12	Empty polyethylene tube placed on sciatic nerve
Supplement (NS)	12	Empty polyethylene tube + Stemregen^®^ (300 mg/kg/day, oral gavage)
Bioceramic (BC)	12	Polyethylene tube filled with AH Plus^®^ Bioceramic Sealer placed on sciatic nerve
Bioceramic + Supplement (BC + NS)	12	Bioceramic Sealer + Stemregen^®^ (300 mg/kg/day, oral gavage)

The table outlines group allocation, sample size, and the specific procedures applied to each group, including bioceramic sealer exposure and systemic Stemregen® administration.

### Surgical procedure

Body weights of all rats were recorded prior to surgery to determine the appropriate anesthesia dosage. General anesthesia was induced by intramuscular injection of ketamine (45 mg/kg; Ketasol 10%, Richter Pharma AG, Wels, Austria) and xylazine (5 mg/kg; Rompun 2%, Bayer Animal Health GmbH, Leverkusen, Germany). Adequate depth of anesthesia was confirmed using the toe pinch reflex.

After anesthesia, the gluteal and thigh regions were shaved, and the animals were positioned in a prone position on the surgical board. The surgical field was disinfected using a povidone–iodine solution (Adeka İlaç, Samsun, Turkey).

A posterolateral longitudinal skin incision was made extending from the right greater trochanter to the lateral condyle of the femur. Blunt dissection was performed between the gluteus superficialis and biceps femoris muscles to expose the right sciatic nerve.

Following exposure of the sciatic nerve, a sterile polyethylene tube (inner diameter: 4 mm; length: 5 mm) was carefully positioned adjacent to the nerve without causing mechanical injury. The tube was stabilized to ensure consistent contact with the neural tissue throughout the experimental period.

In the experimental groups, AH Plus® Bioceramic Sealer (premixed, injectable) was delivered directly into the polyethylene tube using the manufacturer-provided applicator tip to achieve direct contact between the material and the sciatic nerve. In the control group, the tube was left empty. To prevent uncontrolled diffusion of the sealer and to standardize the area of contact, the polyethylene tube was longitudinally sectioned. A sterile paraffin strip was placed between the tube and surrounding soft tissues to isolate the experimental site and restrict material exposure to the sciatic nerve. After placement, the surgical field was irrigated with sterile saline, and the incision was closed using 3-0 silk sutures (Figures 2 and 3).

**Figure 2:**
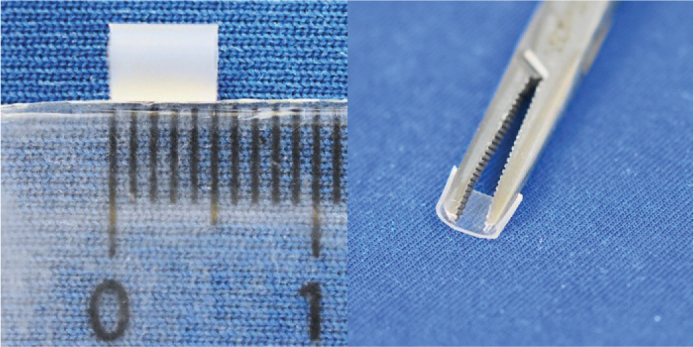
Preparation of the polyethylene tube used for sciatic nerve exposure. The tube was longitudinally sectioned to standardize the contact area and to allow controlled placement of the bioceramic sealer adjacent to the nerve tissue.

**Figure 3:**
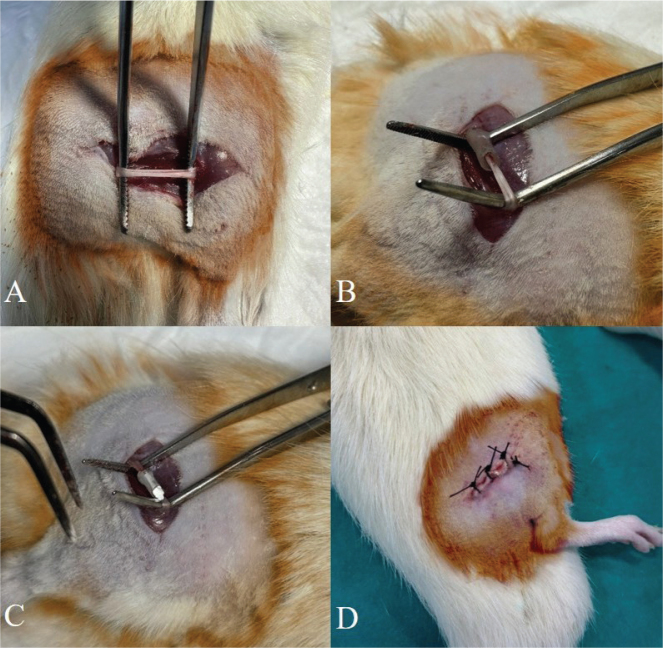
Representative images demonstrating the surgical procedure for sciatic nerve exposure in the rat model. (A) Exposure of the sciatic nerve. (B) Application of an empty polyethylene tube to the sciatic nerve. (C) Application of a polyethylene tube containing root canal sealer to the sciatic nerve. (D) Closure of the surgical site after completion of the procedure.

### Application of nutritional supplement and histopathological analysis

For animals assigned to the nutritional supplement groups, Stemregen® (Biomics™, USA), provided as a powdered formulation in capsules, was administered once daily by oral gavage at a dose of 300 mg/kg, prepared in saline solution, for a period of 15 days after sciatic nerve exposure to assess early histopathological tissue responses [38]. The 15-day observation period was selected to capture early histopathological changes associated with acute neural injury following direct exposure of peripheral nerve tissue to root canal sealers, in accordance with previously established rat sciatic nerve models used for neurotoxicity assessment [20].

At the end of the 15th day of the experiment, all rats were euthanized by gradual exposure to 80% carbon dioxide gas at a gas flow rate of 20% volume/minute. The sciatic nerve was excised, including healthy tissue from the proximal and distal ends of the area where the tube was placed, and then the polyethylene tube applied over the nerve was removed from the nerve tissue (Figure 4).

**Figure 4:**
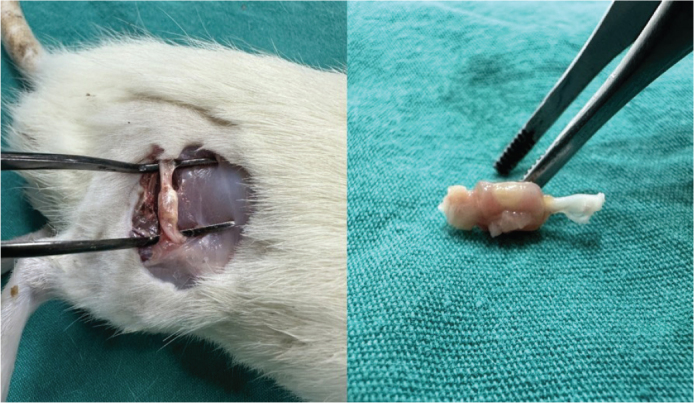
Harvesting of sciatic nerve tissue samples following the experimental period. Representative images show surgical re-exposure of the sciatic nerve and excision of the nerve segment in contact with the polyethylene tube for subsequent histopathological analysis.

The sciatic nerve tissue samples were fixed in 10% buffered formalin solution for 24 hours. The fixed tissues were first washed under running water for 4–8 hours and then passed through a series of alcohol (50–100%) and xylene with increasing concentrations for tissue processing. Subsequently, the tissues were embedded in blocks, and sections 4–5 μm thick were taken using an automatic rotary RM2255 microtome (Leica Biosystems, Wetzlar, Germany). The obtained sections were deparaffinized by being kept in an oven at 60°C for 2 hours. Then the sections were stained with Hematoxylin-Eosin (H&E) and Luxol Fast Blue and mounted with Entellan® (Merck, Darmstadt, Germany). The stained sections were made ready for histological examination.

All samples were examined and photographed with a BX53 trinocular microscope (Olympus Corporation, Tokyo, Japan).

The evaluation of the experimental groups was carried out in three stages. In the first stage, the nerve tissue was evaluated for myelin, axon, and epineurium degeneration. In the second stage, the nerve tissue was evaluated for fibrosis, epineurium, and perineurium thickening. In the third stage, the tissue was semiquantitatively evaluated for lymphocyte infiltration, fibrocyte density, vacuolization, and edema.

Histopathological evaluation was performed by an experienced pathologist who was blinded to the experimental group assignments. In experimental animal models involving surgical nerve exposure and material placement, blinding of the operators performing the interventions is generally not feasible and is not routinely expected, as the applied procedures are inherently distinguishable [20]. Therefore, to minimize the risk of observer bias, all outcome assessments were conducted under blinded conditions, which represents the critical level of blinding for histopathological studies. Semiquantitative histopathological evaluations were performed using predefined scoring criteria detailed in Table 3.

**Table 3:** Semiquantitative histopathological scoring parameters used for the evaluation of sciatic nerve tissue.

Parameter	Description	Scoring
Myelin degeneration	Loss or disruption of myelin sheath	G0 (None) to G5 (Very Severe)
Axonal degeneration	Structural damage or disintegration of axons	G0 to G5
Epineurium degeneration	Degenerative changes in outer nerve sheath	G0 to G5
Perineurial thickening	Structural thickening of perineurium	G0 to G5
Epineurial thickening	Structural thickening of epineurium	G0 to G5
Fibrosis	Presence of fibrotic tissue around or within nerve bundles	G0 to G5
Lymphocytic infiltration	Degree of immune cell infiltration	G0 to G5
Fibrocyte density	Presence of fibrocytes within nerve tissue	G0 to G5
Vacuolization	Intracellular or extracellular vacuole formation	G0 to G5
Edema	Presence of interstitial fluid accumulation	G0 to G5

Semi-quantitative histopathological scoring criteria were defined as: G0 = no pathological findings; G1 = minimal changes; G2 = mild changes; G3 = moderate changes; G4 = marked changes; G5 = severe pathological alterations. Increasing scores reflect greater severity of neural tissue damage.

### Statistical analysis

IBM SPSS Statistics 22 (Armonk, NY: IBM Corp.) program was used for statistical analysis. The conformity of parameters to normal distribution was evaluated with Kolmogorov-Smirnov and Shapiro Wilks tests, and it was determined that the parameters did not show a normal distribution. While evaluating the study data, descriptive statistical methods (minimum, maximum, mean, standard deviation, median) were used, and for comparisons between groups, Kruskal Wallis test and Dunn’s test to determine the group causing the difference were used. Significance was considered at the * p* < 0.05 level.

## Results

Histopathological analysis demonstrated that the BC group exhibited significantly higher degeneration and inflammation scores than the C and NS groups across all evaluated parameters (* p* < 0.05; Tables 4 and 5). In particular, myelin degeneration, axonal degeneration, perineurial and epineurial thickening, fibrosis, edema, and inflammatory cell infiltration were more pronounced in the BC group compared with the other groups.

**Table 4:** Distribution of semiquantitative histopathological scores for sciatic nerve tissue across experimental groups.

	Control group						Supplement group						Bioceramic group						Bioceramic + Supplement group					
	G0	G1	G2	G3	G4	G5	G0	G1	G2	G3	G4	G5	G0	G1	G2	G3	G4	G5	G0	G1	G2	G3	G4	G5
Myelin degeneration	7	5	-	-	-	-	8	4	-	-	-	-	-	-	3	7	2	-	-	4	5	3	-	-
Axonal degeneration	8	4	-	-	-	-	9	3	-	-	-	-	-	3	4	5	-	-	-	6	3	3	-	-
Epineurium degeneration	8	4	-	-	-	-	8	4	-	-	-	-	3	6	3	-	-	-	4	6	2	-	-	-
Fibrosis	9	3	-	-	-	-	9	3	-	-	-	-	-	5	5	2	-	-	-	6	4	2	-	-
Epineurial thickening	10	2	-	-	-	-	10	2	-	-	-	-	-	4	8	-	-	-	-	9	3	-	-	-
Perineurial thickening	9	3	-	-	-	-	9	3	-	-	-	-	-	7	5	-	-	-	-	8	4	-	-	-
Lymphocyte infiltration	7	5	-	-	-	-	8	4	-	-	-	-	-	-	7	4	1	-	-	4	5	3	-	-
Fibrocyte density	8	4	-	-	-	-	8	4	-	-	-	-	-	6	4	2	-	-	-	7	3	2	-	-
Vacuolization	8	4	-	-	-	-	9	3	-	-	-	-	-	5	4	3	-	-	-	7	5	-	-	-
Edema	7	5	-	-	-	-	10	2	-	-	-	-	-	-	3	6	3	-	-	5	6	1	-	-

The table presents the number of specimens corresponding to each grade (G0–G5) for the evaluated parameters. Statistical significance among groups was assessed using the Kruskal–Wallis test followed by Dunn’s post hoc test (* p* < 0.05). G: Grade, G0 (None), G1 (Mild), G2 (Moderate), G3 (Significant), G4 (Severe), and G5 (Very Severe).

**Table 5:** Comparison of experimental groups based on semiquantitative histopathological scores of sciatic nerve tissue.

Histopathological Evaluated Parameters	Control group (a)			Supplement group (b)			Bioceramic group (c)			Bioceramic + Supplement group (d)			* P*
	Min	Med	Max	Min	Med	Max	Min	Med	Max	Min	Med	Max
Myelin degeneration	0	0.42 ± 0.51 (0) **(a–b)**	1	0	0.33 ± 0.49 (0) (b–c*)(b–d*)	1	2	2.92 ± 0.67 (3) (a–c*)(b–c*)(c–d)	4	1	1.92 ± 0.79 (2) (a–d*)(b–d*)	3	**0.001**
Axonal degeneration	0	0.33 ± 0.49 (0) **(a–b)**	1	0	0.25 ± 0.45 (0) (b–c*)(b–d*)	1	1	2.17 ± 0.83 (2) (a–c*)(b–c*)(c–d)	3	1	1.75 ± 0.87 (1.5) (a–d*)(b–d*)	3	**0.001**
Epineurium degeneration	0	0.33 ± 0.49 (0) **(a–b)**	1	0	0.33 ± 0.49 (0) (b–c*)	1	0	1.0 ± 0.74 (1) (a–c*)(b–c*)(c–d)	2	0	0.83 ± 0.72 (1)	2	**0.032**
Fibrosis	0	0.25 ± 0.45 (0) **(a–b)**	1	0	0.25 ± 0.45 (0) (b–c*)(b–d*)	1	1	1.75 ± 0.75 (2) (a–c*)(b–c*)(c–d)	3	1	1.67 ± 0.78 (1.5) (a–d*)(b–d*)	3	**0.001**
Epineurial thickening	0	0.17 ± 0.39 (0) **(a–b)**	1	0	0.17 ± 0.39 (0) (b–c*)(b–d*)	1	1	1.67 ± 0.49 (2) (a–c*)(b–c*)(c–d)	2	1	1.25 ± 0.45 (1) (a–d*)(b–d*)	2	**0.001**
Perineurial thickening	0	0.25 ± 0.45 (0) **(a–b)**	1	0	0.25 ± 0.45 (0) (b–c*)(b–d*)	1	1	1.42 ± 0.51 (1) (a–c*)(b–c*)(c–d)	2	1	1.33 ± 0.49 (1) (a–d*)(b–d*)	2	**0.001**
Lymphocyte infiltration	0	0.42 ± 0.51 (0) **(a–b)**	1	0	0.33 ± 0.49 (0) (b–c*)(b–d*)	1	2	2.5 ± 0.67 (2) (a–c*)(b–c*)(c–d)	4	1	1.92 ± 0.79 (2) (a–d*)(b–d*)	3	**0.001**
Fibrocyte density	0	0.33 ± 0.49 (0) **(a–b)**	1	0	0.33 ± 0.49 (0) (b–c*)(b–d*)	1	1	1.67 ± 0.78 (1.5) (a–c*)(b–c*)(c–d)	3	1	1.58 ± 0.79 (1) (a–d*)(b–d*)	3	**0.001**
Vacuolization	0	0.33 ± 0.49 (0) **(a–b)**	1	0	0.25 ± 0.45 (0) (b–c*)(b–d*)	1	1	1.83 ± 0.83 (2) (a–c*)(b–c*)(c–d)	3	1	1.42 ± 0.51 (1) (a–d*)(b–d*)	2	**0.001**
Edema	0	0.42 ± 0.51 (0) **(a–b)**	1	0	0.17 ± 0.39 (0) (b–c*)(b–d*)	1	2	3.0 ± 0.74 (3) (a–c*)(b–c*)(c–d)	4	1	1.67 ± 0.65 (1) (a–d*)(b–d*)	3	**0.001**

Data are presented as minimum, median, and maximum values for each parameter across 12 rats per group. Intergroup comparisons were performed using the Kruskal–Wallis test followed by Dunn’s post hoc analysis, with statistically significant differences indicated (p < 0.05). P: Significance level, Min: Minimum, Med: Median, Max: Maximum, a: Control group, b: Supplement group, c: Bioceramic group, d: Bioceramic + Supplement group. P values shown in the final column represent the overall Kruskal–Wallis test results for each parameter. Pairwise group comparisons were subsequently performed using Dunn’s post hoc test. Comparisons marked with an asterisk (*) indicate statistically significant differences (p < 0.05), whereas comparisons without an asterisk were not statistically significant. Bold P values indicate statistically significant overall Kruskal–Wallis test results (p < 0.05).

The BC + NS group exhibited lower median scores across all evaluated histopathological parameters compared with the BC group. This reduction was most evident for axonal degeneration, fibrosis, and inflammatory cell infiltration; however, these differences did not reach statistical significance (* p* > 0.05; Tables 4 and 5).

The NS and C groups exhibited minimal histopathological alterations, with scores predominantly ranging from grade 0 to grade 1 across all evaluated parameters. Detailed semiquantitative scoring results are presented in Tables 4 and 5, and representative histological findings are illustrated in Figures 5 and 6.

**Figure 5:**
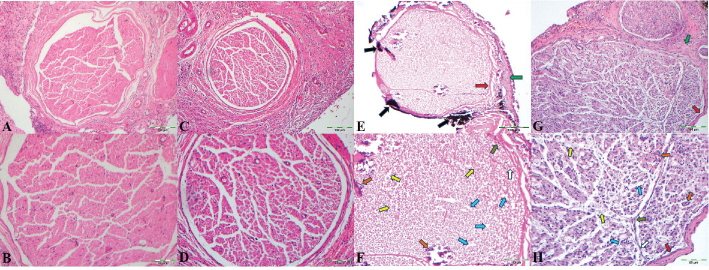
Representative hematoxylin and eosin–stained sections of sciatic nerve tissue from different experimental groups. (A, B) Control group at 10× and 20× magnification, respectively. (C, D) Nutritional Supplement group at 10× and 20× magnification, respectively. (E, F) Bioceramic group at 10× and 20 magnification, respectively, exhibiting marked pathological changes (red arrow: perineurial thickening; green arrow: epineurial thickening and degeneration; black arrow: sealer residue; orange arrow: lymphocyte infiltration; white arrow: increased fibrocyte density; yellow arrow: vacuolization; dark green arrow: fibrosis; blue arrow: edema). (G, H) Bioceramic + Nutritional Supplement group at 10× and 20× magnification, respectively (red arrow: thickening in the perineurium, green arrow: thickening and degeneration in the epineurium, orange arrow: lymphocyte infiltration, white arrow: fibrocyte density, yellow arrow: vacuolization, dark green arrow: fibrosis, blue arrow: edema).

**Figure 6:**
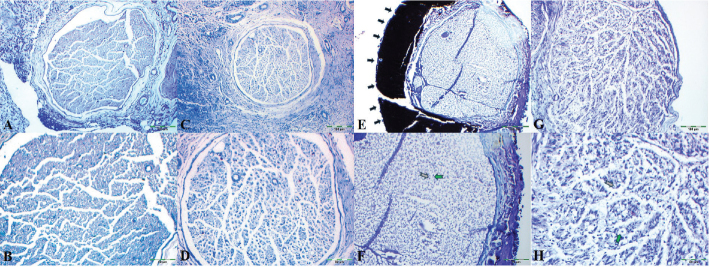
Representative Luxol Fast Blue-stained sections of sciatic nerve tissue. (A, B) Control group at 10× and 20× magnification, respectively. (C, D) Nutritional Supplement group at 10× and 20× magnification, respectively. (E, F) Bioceramic group at 10× and 20× magnification, respectively (black arrow: sealer residue; green arrow: myelin degeneration; gray arrow: axonal degeneration). (G, H) Bioceramic + Nutritional Supplement group at 10× and 20× magnification, respectively (green arrow: myelin degeneration; gray arrow: axonal degeneration).

## Discussion

The present study demonstrated that direct exposure of the rat sciatic nerve to a bioceramic root canal sealer was associated with pronounced histopathological degeneration and inflammatory changes. Although the Stemregen®-treated group demonstrated consistently lower histopathological scores, the differences did not reach statistical significance. Accordingly, the null hypothesis was partially rejected, as the bioceramic sealer induced significant neural tissue alterations, whereas no statistically significant modulatory effect of Stemregen® was observed.

### Neurotoxic effects of bioceramic sealer

The present findings are consistent with previous experimental work using a rat sciatic nerve model, which has shown that direct exposure to root canal sealers may induce histopathological nerve alterations [20]. In the present study, application of the bioceramic sealer was associated with marked neural changes, including myelin disruption, axonal injury, perineurial thickening, and inflammatory cell infiltration. Similar adverse neural responses have been reported when endodontic sealers are extruded in close proximity to neural structures [5, 6].

Although calcium silicate–based sealers are widely regarded as biocompatible based on in vitro investigations [16–18], such experimental conditions do not adequately reflect the complex biological environment encountered during direct nerve contact in vivo. In line with clinical case reports and experimental studies describing neural complications following sealer extrusion into neurovascular spaces [19, 20], the present in vivo findings suggest that direct exposure may be associated with unfavorable neural tissue responses. These observations underscore the need for clinical caution when bioceramic sealers are used in anatomical regions adjacent to critical neural structures, such as the inferior alveolar nerve.

One potential mechanism contributing to the observed neural tissue alterations is the alkaline environment generated during the setting reaction of tricalcium silicate–based materials, which has been suggested to affect nerve membrane stability, disrupt calcium homeostasis, and activate cellular stress pathways in neural tissues [18–20]. In addition, formulation-dependent differences among calcium silicate–based sealers may influence their interaction with surrounding tissues. Variations in setting behavior and physical stabilization between premixed and powder–liquid formulations may affect the duration and extent of material–tissue contact under in vivo conditions, potentially contributing to the variability in biological responses reported across experimental studies [16–20].

In addition, formulation additives such as dimethyl sulfoxide (DMSO) and lithium carbonate may contribute to neural tissue responses following direct sealer exposure. DMSO has been reported to increase cell membrane permeability, potentially facilitating the diffusion of reactive components into neural tissue and increasing cytotoxic susceptibility [39, 40]. Although lithium carbonate is widely used therapeutically in neuropsychiatric disorders, evidence regarding its effects on myelin integrity is derived predominantly from central nervous system models, and data on peripheral nerve tissue remain limited and inconclusive [23, 24]. Furthermore, diffusion of sealer-derived chemical components toward adjacent neural tissue may alter local cellular homeostasis and inflammatory signaling, representing an additional mechanism underlying the observed histopathological alterations.

Mechanical factors related to the experimental setup may also have contributed to the observed neural tissue changes. In particular, pressure exerted by the polyethylene tube may impair local blood flow, potentially leading to ischemic or hypoxic conditions and secondary inflammatory responses in nerve tissue [41]. Surgical manipulation itself may likewise induce a degree of inflammatory response, which could explain the mild histopathological changes observed in the Control and Nutritional Supplement groups and may have augmented tissue alterations in the Bioceramic and Bioceramic + Nutritional Supplement groups [42]. However, as all experimental groups were subjected to identical surgical procedures, mechanical and surgical factors alone are unlikely to account for the observed group-dependent differences.

From a clinical perspective, these findings may be particularly relevant for posterior mandibular regions, where the close anatomical relationship between root apices and the mandibular canal increases the risk of sealer extrusion toward the inferior alveolar nerve [3, 4, 10]. The present experimental model suggests that localized neural exposure to bioceramic sealers may be associated with histopathological alterations even over a relatively short observation period. Nevertheless, extrapolation to clinical practice should be approached with caution, as the experimental conditions do not fully replicate the complexity of human periapical tissues or routine treatment scenarios.

A sciatic nerve model was selected due to its large caliber, well-defined anatomy, and ease of surgical access, allowing reproducible placement of test materials and standardized histological assessment. Although anatomically distinct, its mixed motor–sensory composition shares functional characteristics with the inferior alveolar nerve, and it has therefore been widely used as a surrogate model for peripheral neurotoxicity evaluation [43].

Moreover, the direct-contact application employed in this study represents a worst-case experimental scenario rather than typical clinical conditions of apical extrusion, where surrounding periapical tissues may partially buffer or dilute material exposure. Thus, while this model provides valuable insight into potential biological responses to direct neural contact, extrapolation to clinical practice should be undertaken with caution.

### Histopathological findings associated with Stemregen® supplementation

The BC + NS group demonstrated a consistent downward trend across all evaluated inflammation and degeneration parameters compared with the Bioceramic-only group, with the most notable reductions observed in axonal degeneration, fibrocyte density, and vacuolization; however, these differences did not reach statistical significance (* p* > 0.05). The lack of statistical significance may be related to the acute duration of the experimental model or to a modest magnitude of the supplement-associated effect, as suggested in previous experimental studies [26, 28]. Although the BC + NS group demonstrated numerically lower histopathological scores than the BC group, no statistically significant differences were detected. Therefore, the present findings do not provide evidence of a protective or modulatory effect of Stemregen® under the conditions of this study. The observed numerical differences should be interpreted cautiously and may reflect biological variability rather than a true treatment-related effect.

According to the manufacturer, Stemregen® is a commercially available multi-ingredient dietary supplement formulated to modulate the systemic immune environment and promote endogenous stem cell mobilization from the bone marrow. Its botanical constituents include sea buckthorn, Panax notoginseng, fucoidan, Aloe vera, and algae-derived compounds, several of which – such as Panax notoginseng–derived saponins, Aloe vera–based compounds, beta-glucans, and algae-derived polysaccharides – have demonstrated anti-inflammatory, antioxidant, or immunomodulatory properties in experimental models when evaluated individually [29–37, 44, 45].

Stemregen® is a dietary supplement that has been marketed as a stem cell–mobilizing formulation. Although several of its individual constituents have demonstrated anti-inflammatory or antioxidant properties in experimental settings, evidence regarding the biological activity of the combined formulation remains limited. Accordingly, it was included on an exploratory basis to determine whether such a multi-targeted systemic intervention could influence early histopathological responses following nerve exposure. Direct attribution of the observed histopathological trends to stem cell mobilization or synergistic anti-inflammatory mechanisms therefore remains speculative, highlighting the need for further studies incorporating longer observation periods, functional outcome measures, and direct assessment of stem cell–related markers.

### Study limitations and future directions

Several limitations should be considered when interpreting the present findings. First, the analysis was limited to semiquantitative histopathological assessment, which does not capture molecular or functional aspects of nerve injury. Although histopathology provides essential information on structural damage and inflammation, it does not directly reflect functional neural integrity. Furthermore, the presence of histopathological alterations should not be interpreted as direct evidence of clinically relevant neural dysfunction, as structural tissue changes may not necessarily correspond to measurable sensory, motor, or electrophysiological deficits. Therefore, the translational implications of the present findings remain limited until supported by electrophysiological, behavioral, and other functional assessments in future studies. In addition, semiquantitative histopathological scoring inherently involves a degree of observer-dependent interpretation and may be less sensitive than quantitative morphometric, molecular, or digital image–based analyses for detecting subtle tissue alterations.

Second, only one bioceramic root canal sealer was evaluated. At the time the study was designed and conducted, AH Plus® Bioceramic Sealer was commercially available and in clinical use. Importantly, key physicochemical characteristics of this material – such as its tricalcium silicate–based composition and alkaline setting reaction – are common to many calcium silicate–based sealers currently used in clinical practice, supporting the potential relevance of the present findings beyond a single product [18].

Third, although polyethylene tubes were used to standardize material placement, the degree of mechanical compression applied to the sciatic nerve was not quantitatively assessed. Thus, the contribution of compression-related ischemia to the observed histopathological changes cannot be fully excluded [41]. However, identical tubes were placed adjacent to the sciatic nerve in all experimental groups, indicating that mechanical pressure alone is unlikely to account for the pronounced degeneration and inflammatory changes observed specifically in the Bioceramic-treated groups. Future studies should incorporate standardized pressure measurements or alternative delivery systems to further distinguish mechanical from material-related effects.

Another limitation relates to the experimental model itself. Although the rat sciatic nerve model enables standardized assessment of direct sealer–nerve interactions, it does not fully reproduce the clinical scenario of root canal sealer extrusion into periapical tissues or the mandibular canal. Therefore, the findings should be interpreted as representing the consequences of direct peripheral nerve contact with the material rather than a true clinical sealer extrusion model.

Future investigations should incorporate longer observation periods to assess delayed tissue responses and potential regenerative processes. The present 15-day observation period was designed to evaluate acute histopathological responses and therefore does not permit assessment of long-term nerve regeneration, chronic inflammatory modulation, remyelination, or functional recovery. In addition, future studies should investigate the local and systemic migration of chemical elements released from bioceramic sealers using analytical techniques such as mass spectrometry, including assessment of accumulation in peripheral nerve tissue and distant organs such as the brain. Comparative studies involving different bioceramic sealers and stem cell–modulating formulations may also help distinguish material-specific from formulation-related effects.

The absence of statistically significant differences between the BC and BC + NS groups may, at least in part, be related to the relatively short supplementation period. Although a 15-day duration was selected to evaluate early histopathological responses, longer treatment regimens may help clarify whether any biologically meaningful effects emerge over time.

From a clinical perspective, extrapolation of these findings should be approached with caution. Although direct contact between root canal sealers and neural tissue is uncommon in routine practice, accidental extrusion into adjacent neurovascular structures – particularly in anatomically vulnerable regions such as the mandibular canal – can occur. These findings underscore the importance of meticulous working length determination and controlled obturation techniques, especially when using materials with high alkalinity or reactive excipients.

## Conclusions

Within the limitations of this in vivo rat model, direct exposure of peripheral nerve tissue to AH Plus® Bioceramic Sealer was associated with pronounced histopathological alterations, characterized by increased neural degeneration and inflammatory changes. Systemic administration of Stemregen® was not associated with statistically significant improvements in histopathological outcomes compared with the bioceramic-only group.

Taken together, these findings underscore the importance of minimizing sealer extrusion, particularly in anatomically sensitive regions, and highlight the need for further preclinical and translational studies to better characterize neural tissue responses to calcium silicate–based sealers and to clarify the potential role of regenerative adjunctive strategies. Because functional neural outcomes were not evaluated, the present findings should be interpreted as evidence of structural histopathological alterations rather than confirmed neural dysfunction. These conclusions are derived primarily from semiquantitative histopathological assessments, and functional or molecular outcomes were beyond the scope of the present study. Longer-term studies are required to determine whether the observed histopathological alterations persist, resolve, or progress over time.

## Data Availability

The data that support the findings of this study are available from the corresponding author upon reasonable request. The data are not publicly available due to privacy or ethical restrictions.
